# Supplementary material for: Variant in the synaptonemal complex protein SYCE2 associates with pregnancy loss through effect on recombination
Source: Nat Struct Mol Biol. 2024 Jan 29;31(4):710–6. doi: 10.1038/s41594-023-01209-y (PMC11026158; doi:10.1038/s41594-023-01209-y)
Supplement: Supplementary file 1 — Supplementary Tables 1–8 and Note. [file 41594_2023_1209_MOESM1_ESM.pdf]

nature structural & molecular biology

Article

https://doi.org/10.1038/s41594-023-01209-y

Variant in the synaptonemal complex protein SYCE2 associates with pregnancy loss through effect on recombination

In the format provided by the authors and unedited

Table of contents:

Supplementary Tables 2

Supplementary Note 9

- Quality control of the SYCE2:p.His89Tyr variant rs189296436 9
- Comparison with Laisk et al. study 9
- DBDS genomics consortium 11

Supplementary Table 1. Pregnancy loss cases

		N					
	Definition	Iceland	Denmark	UK	USA	Finland	Total
Spontaneous abortion	ICD10:O03; ICD9;634; ICD8:643	4,306	13,769	8,345	1,165	15,073	42,658
Recurrent pregnancy loss	ICD10:N96,O262; ICD9:6298; ICD8:6430	171	1,197	85	321	479	2,253
Missed abortion	ICD10:O021; ICD9:632; ICD8:634,6451	3,860	9,875	2,165	921	-	16,821
Self report	Self report	20,128	-	47,883	-	-	68,011
Total unique cases		26,956	21,978	48,954	1,800	15,073	114,761
Controls	Females	156,495	59,626	183,598	29,923	135,962	565,604

Supplementary Table 2. Association results for SYCE2:p.His89Tyr in individual populations We used logistic regression to test for association between genotype count as predictor and diseases status as outcome in each dataset, adjusting for covariates such as YOB, sex and populations structure. P values are two-sided without Bonferroni correction. The table includes MAF in each dataset and the imputation info as an estimate of the imputation quality. The combined results, P value and OR, are obtained from the fixed effect inverse variance weighted meta-analysis of results from individual datasets.

Dataset	P	OR	95% CI	MAF %	Info
Iceland	0.0237	1.12	(1.01-1.23)	1.27	1.00
Denmark	4.68×10^{-5}	1.32	(1.15-1.50)	0.72	0.97
UK	5.52×10^{-7}	1.27	(1.16-1.40)	0.51	0.99
USA	0.533	1.16	(0.73-1.82)	0.58	0.96
Finland	0.00879	1.42	(1.09-1.85)	0.18	1.00
Combined*	6.60×10^{-12}	1.22	(1.16-1.30)		

*Heterogeneity P-value (P_{het}) 0.14

Supplementary Table 3. Association of SYCE2:p.His89Tyr with individual phenotypes included in the definition of pregnancy loss

The table includes, for each trait, the total number of cases and controls in the meta-analysis, and the OR, P-value and 95% CI from a fixed effect inverse variance weighted meta-analysis of results for individuals cohorts included for each trait. P values are two-sided without Bonferroni correction.

Trait	cases	controls	P	OR	95%CI
Spontaneous abortion	42,658	551,395	2.90×10^{-7}	1.29	(1.17-1.43)
Recurrent pregnancy loss	2,253	515,923	0.094	1.37	(0.95-2.00)
Missed abortion	16,821	409,212	0.0068	1.20	(1.05-1.37)
Pregnancy loss ICD codes combined	55,961	551,285	6.10×10^{-8}	1.26	(1.16-1.38)
Pregnancy loss self reported	68,011	212,160	1.70×10^{-6}	1.20	(1.11-1.29)
Pregnancy loss	114,761	565,604	6.60×10^{-12}	1.22	(1.16-1.30)

Supplementary Table 4. Variants previously reported to associate with pregnancy loss phenotypes do not associate with corresponding phenotypes in current study. Four variants were reported [PMID: 33239672] to associate with pregnancy loss (rs146350366) or recurrent pregnancy loss (rs7859844, rs143445068, rs183453668). No data is available for rs183453668 as it did not pass quality filters in the deCODE whole genome sequencing data and it is not available in finngen (r8.finngen.fi/). We also note that it did not pass quality filters in gnomAD (gnomad.broadinstitute.org). The table shows association results, P value, OR and 95% CI, from logistic regression for the other three variants in each of the datasets included, and the combined results, from a fixed effect inverse variance weighted meta-analysis of results for individual datasets. Effect estimates are presented for the minor allele. P values are two-sided without Bonferroni correction.

	rs146350366 MAF=1.02% Pregnancy loss				rs7859844 MAF =6.57% Recurrent pregnancy loss				rs143445068 MAF=0.90% Recurrent pregnancy loss			
Population	Pvalue	OR	95%CI	MAF %	Pvalue	OR	95%CI	MAF %	Pvalue	OR	95%CI	MAF %
Iceland	0.19	0.75	(0.49-1.15)	0.08	0.71	1.09	(0.69-1.72)	7.52	0.51	0.02	(0.00-2266.51)	0.07
UK	0.0047	0.84	(0.74-0.95)	0.34	0.12	1.55	(0.89-2.69)	6.32	0.88	1.15	(0.19-7.06)	0.81
USA	0.98	0.99	(0.45-2.17)	0.49	0.92	0.98	(0.66-1.45)	6.43	0.17	1.77	(0.78-4.00)	0.73
Denmark	0.16	1.10	(0.96-1.26)	0.68	0.40	1.08	(0.90-1.29)	5.91	0.84	1.06	(0.60-1.87)	0.66
Finland	0.38	0.97	(0.91-1.04)	2.83	0.64	0.95	(0.77-1.18)	11.04	0.50	1.33	(0.58-3.05)	0.63
Meta	0.12	0.96	(0.91-1.01)		0.49	1.04	(0.93-1.16)		0.26	1.25	(0.85-1.84)	

Supplementary Table 5. Effects of SYCE2:p.His89Tyr on the average telomere distance of maternal crossovers in offspring of carriers. The table shows the association results with 95% CI. Effects are listed in units of Mb and standard deviation (SD). Association analysis was performed with linear regression under an additive genetic model, where the phenotype corresponds to the average telomere distance of crossovers in all offspring. CIs are inferred from P-values, which are computed with a two-sided t-test and unadjusted for multiple comparisons. The P-values shown in column 4 pertain to the association results in SD units. The singleton results in the second half of the table show the association results when we restrict to probands with a single crossover chromosome.

Chromosome	Effect on telomere distance		P-value
	Mb (95% CI)	SD (95% CI)	
**All crossovers**			
chr1	−4.6 (−5.5 to −3.6)	−0.33 (−0.4 to −0.26)	9.70×10^{-20}
chr2	−6 (−7.1 to −5)	−0.41 (−0.48 to −0.34)	1.60×10^{-30}
chr3	−3.4 (−4.2 to −2.5)	−0.28 (−0.35 to −0.21)	7.20×10^{-15}
chr4	−3.4 (−4.3 to −2.6)	−0.26 (−0.33 to −0.19)	8.20×10^{-13}
chr5	−3.3 (−4.2 to −2.5)	−0.27 (−0.34 to −0.19)	2.40×10^{-13}
chr6	−2.7 (−3.5 to −1.9)	−0.24 (−0.31 to −0.17)	1.80×10^{-11}
chr7	−3.1 (−3.9 to −2.3)	−0.28 (−0.36 to −0.21)	1.40×10^{-14}
chr8	−2.4 (−3.2 to −1.7)	−0.23 (−0.3 to −0.16)	4.50×10^{-10}
chr9	−2.4 (−3.1 to −1.7)	−0.24 (−0.31 to −0.16)	1.30×10^{-10}
chr10	−1.1 (−1.8 to −0.42)	−0.11 (−0.19 to −0.042)	0.0019
chr11	−1.2 (−2 to −0.51)	−0.12 (−0.19 to −0.048)	0.0011
chr12	−1.4 (−2.1 to −0.7)	−0.13 (−0.2 to −0.057)	0.00041
chr13	−1.2 (−1.8 to −0.61)	−0.14 (−0.22 to −0.073)	8.60×10^{-5}
chr14	−0.94 (−1.6 to −0.33)	−0.12 (−0.19 to −0.045)	0.0015
chr15	−0.72 (−1.3 to −0.12)	−0.073 (−0.15 to 0.00069)	0.052
chr16	−1.1 (−1.6 to −0.61)	−0.16 (−0.23 to −0.086)	1.80×10^{-5}
chr17	−0.18 (−0.65 to 0.29)	−0.023 (−0.097 to 0.051)	0.54
chr18	−0.58 (−1.1 to −0.099)	−0.07 (−0.14 to 0.0036)	0.062
chr19	−0.3 (−0.67 to 0.066)	−0.054 (−0.13 to 0.02)	0.15
chr20	−0.067 (−0.41 to 0.28)	0.004 (−0.071 to 0.079)	0.92
chr21	−0.44 (−0.79 to −0.09)	−0.095 (−0.17 to −0.016)	0.019
chr22	−0.29 (−0.69 to 0.12)	−0.04 (−0.12 to 0.037)	0.31
**Singletons**			
chr1	−6.3 (−11 to −1.6)	−0.14 (−0.29 to 0.014)	0.076
chr2	−12 (−16 to −7)	−0.36 (−0.5 to −0.21)	1.00×10^{-6}
chr3	−4.5 (−7.6 to −1.4)	−0.22 (−0.35 to −0.096)	0.00057
chr4	−8.4 (−11 to −5.4)	−0.32 (−0.44 to −0.2)	1.90×10^{-7}
chr5	−6.4 (−9.2 to −3.7)	−0.19 (−0.31 to −0.078)	0.001
chr6	−5.3 (−7.9 to −2.8)	−0.23 (−0.35 to −0.11)	0.00024
chr7	−4.9 (−7.1 to −2.6)	−0.26 (−0.37 to −0.14)	1.10×10^{-5}
chr8	−3 (−5 to −1.1)	−0.17 (−0.27 to −0.058)	0.0024
chr9	−3.1 (−4.8 to −1.3)	−0.2 (−0.31 to −0.092)	0.00027
chr10	−1.4 (−3.3 to 0.5)	−0.078 (−0.19 to 0.031)	0.16
chr11	−1.6 (−3.3 to 0.15)	−0.075 (−0.18 to 0.028)	0.16
chr12	−2 (−3.9 to −0.18)	−0.071 (−0.18 to 0.037)	0.2
chr13	−1.6 (−2.7 to −0.37)	−0.12 (−0.21 to −0.028)	0.011
chr14	−1.3 (−2.4 to −0.22)	−0.12 (−0.21 to −0.026)	0.011
chr15	−1.4 (−2.5 to −0.32)	−0.11 (−0.2 to −0.016)	0.021
chr16	−1.3 (−2.2 to −0.35)	−0.12 (−0.22 to −0.029)	0.01
chr17	0.55 (−0.4 to 1.5)	0.07 (−0.026 to 0.17)	0.15
chr18	−0.73 (−1.6 to 0.15)	−0.054 (−0.15 to 0.039)	0.26
chr19	−0.51 (−1.1 to 0.08)	−0.075 (−0.16 to 0.014)	0.1
chr20	−0.28 (−0.81 to 0.26)	−0.04 (−0.13 to 0.05)	0.38
chr21	−0.43 (−0.85 to −0.0086)	−0.075 (−0.16 to 0.01)	0.085
chr22	−0.33 (−0.84 to 0.18)	−0.034 (−0.12 to 0.051)	0.43

Supplementary Table 6. Effects of SYCE2.pHis89Tyr on the average per-chromosome maternal recombination rate in offspring of carriers. The table shows the association results with 95% CI. Effects are listed in units of cM and standard deviation (SD). Association analysis was performed with linear regression under an additive genetic model, where the phenotype corresponds to the average of the per-chromosome recombination rate in all offspring. CIs are inferred from P-values, which are computed with a two-sided t-test and not adjusted for multiple comparisons. The P-values shown in column 4 pertain to the association results in SD units. Recombination rates are corrected for maternal age before the carrier effects are computed.

Chromosome	Effect on the average per-chromosome recombination rate		P-value
	cM (95% CI)	SD (95% CI)	
**chr1**	−24 (−32 to −15)	−0.19 (−0.26 to −0.12)	$1.60{\times}10^{-7}$
**chr2**	−31 (−39 to −23)	−0.25 (−0.32 to −0.18)	$4.80{\times}10^{-12}$
**chr3**	−15 (−23 to −7.7)	−0.14 (−0.21 to −0.07)	0.0001
**chr4**	−16 (−24 to −9)	−0.12 (−0.19 to −0.045)	0.0013
**chr5**	−11 (−18 to −4)	−0.096 (−0.17 to −0.025)	0.0079
**chr6**	−5.7 (−13 to 1.1)	−0.043 (−0.11 to 0.028)	0.23
**chr7**	−6.4 (−13 to 0.19)	−0.071 (−0.14 to −0.00041)	0.049
**chr8**	−4.2 (−11 to 2.1)	−0.034 (−0.1 to 0.036)	0.34
**chr9**	0.27 (−5.8 to 6.4)	0.032 (−0.039 to 0.1)	0.38
**chr10**	0.085 (−6.3 to 6.5)	0.02 (−0.051 to 0.091)	0.58
**chr11**	5 (−1.1 to 11)	0.05 (−0.022 to 0.12)	0.17
**chr12**	0.96 (−5.2 to 7.2)	0.01 (−0.064 to 0.084)	0.79
**chr13**	−0.75 (−6.1 to 4.6)	−0.002 (−0.061 to 0.057)	0.95
**chr14**	−2.3 (−7.3 to 2.7)	−0.022 (−0.092 to 0.048)	0.54
**chr15**	−6.5 (−12 to −1.4)	−0.078 (−0.15 to −0.0066)	0.032
**chr16**	4.5 (−0.78 to 9.7)	0.075 (0.0051 to 0.14)	0.036
**chr17**	4.8 (−0.51 to 10)	0.065 (−0.0059 to 0.14)	0.072
**chr18**	5.5 (0.43 to 10)	0.083 (0.012 to 0.15)	0.022
**chr19**	9.5 (4.9 to 14)	0.15 (0.083 to 0.22)	$2.00{\times}10^{-5}$
**chr20**	8.2 (3.5 to 13)	0.13 (0.056 to 0.2)	0.00047
**chr21**	0.43 (−3.3 to 4.2)	0.034 (−0.037 to 0.1)	0.35
**chr22**	4.6 (0.75 to 8.4)	0.09 (0.02 to 0.16)	0.012

Supplementary Table 7 Effect of SYCE2:p.His89Tyr on number of children

The table includes the number of mothers included for each dataset and their mean (and 95% CI) number of children broken down by genotype status of SYCE2:p.His89Tyr. The association between genotype count and number of children, in each dataset, was tested assuming a Conway-Maxwell Poisson distribution implemented in the glmmTMB package in R, both for the additive and recessive genotype model, and the resulting P values and OR combined using a fixed effect inverse variance weighted meta-analysis. All P values are two-sided.

	Mean number of children by SYCE2:p.His89Tyr genotype of mothers						Association between SYCE2:p.His89Tyr genotype and number of children					
	Non-carriers		Heterozygotes		Homozygotes		Additive model			Recessive model		
Dataset	N mothers*	Mean N children (95%CI)	N mothers	Mean N children (95%CI)	N mothers	Mean N children (95%CI)	OR (95%CI)	P	P_{het}	OR (95%CI)	P	P_{het}
Iceland	70,952	2.782 (2.769,2.794)	1814	2.742 (2.665,2.818)	13	2 (1.231,2.768)	0.997	0.88		0.749	0.082	
UK	229,658	1.819 (1.813,1.824)	2373	1.812 (1.757,1.866)	5	2 (0.760,3.239)	0.985	0.21		1.037	0.9	
Denmark	26,586	1.857 (1.841,1.874)	397	1.869 (1.734,2.004)	0	-	1.005	0.87		-	-	
Meta							0.990 (0.971-1.009)	0.30	0.76	0.812 (0.612-1.077)	0.15	0.33

*Icelandic women born 1918-1983, Danish women born 1957-1973 and all women from UK Biobank were included in the analysis

Supplementary Table 8. Association results for pregnancy loss on variants that have previously been shown to associate with crossover recombination data. Three of the 47 original variants have shifted from earlier variant-call freezes [PMID: 30679340]. The new variants are in the same genes as the replaced ones, and with similar effects and p-values for the strongest associating phenotypes. Thus, chr11:66843338 (freq: 8.0%, associating with RH(joint), effect: 0.075, p-value: 2.7×10^{-11}) is shifted to chr11:66843352 (freq: 8.0%, associating with RH(joint), effect: 0.071, p-value: 2.3×10^{-9}), chr20:1226069 (freq: 47.6%, associating with TD(paternal), effect: −0.156, p-value: 1.6×10^{-80}) is shifted to chr20:1226073 (freq: 47.5%, associating with TD(paternal), effect: −0.154, p-value: 1.2×10^{-64}), and chrX:135864034 (freq: 26.2%, associating with RR(paternal), effect: 0.071, p-value: 2.1×10^{-18}) is shifted to chrX:135867536 (freq: 35.0%, associating with RR(paternal), effect: 0.073, p-value: 4.3×10^{-22}). MAF refers to minor allele frequency in the Icelandic cohort. The P values and OR are from a fixed effect inverse variance weighted meta-analysis of association results for individual datasets of pregnancy loss on genotype count, calculated using logistic regression and adjusting for covariates such as YOB, sex and population structure. P values are two-sided without Bonferroni correction *Impact: ms, missense; sp, splice region (exon/intron boundary, ranging from +8 bp into intron to 2 bp into exon); ns, nonsense; fs, frameshift; up/down, 5 kb up- or downstream of the transcription start site; syn, synonymous; int, intronic.

SNO	Chr	Pos	Gene/*Impact	Alteration	MAF(%)	OR	P-value
**Coding and splice region variants**							
1	chr4	682038	SLC49A3/ms	NP_001281270.1:p.Ala535Thr	0.2	1.087	0.502
2	chr10	133560087	SYCE1/sp	NM_001143763.1:c.136+4G>A	9.3	0.997	0.731
3	chr14	20316559	CCNB1IP1/sp	NM_021178.5:c.-36C>A	48.4	0.993	0.149
4	chr16	1844960	MEIOB/ms	NP_001157032.1:p.Ile261Thr	15.6	0.988	0.051
5	chr17	38945127	FBXO47/ms	NP_001008777.2:p.Gln209Arg	6.8	0.999	0.878
6	chrX	104040193	H2BFM/ns	NP_001157888.1:p.Gln73Ter	46.5	0.996	0.463
7	chrX	135867536	CT45A9/int	NM_001291540.2:c.169+357G>C	35.0	0.997	0.623
8	chr1	75880138	MSH4/ms	NP_002431.2:p.Tyr589Cys	1.6	0.982	0.373
9	chr1	91394244	HFM1/ms	NP_001017975.4:p.Ser115Pro	29.4	0.993	0.2
10	chr4	1093477	RNF212/ms	NP_001180247.1:p.Ile262Val	22.8	0.985	0.00845
11	chr14	60437039	C14orf39/ms	NP_777638.3:p.Leu524Phe	31.3	0.985	0.00551
12	chr17	45983409	MAPT/ms	NP_001116538.2:p.Pro202Leu	18.2	1.001	0.855
13	chr20	1230003	RAD21L1/ms	NP_001130038.2:p.Cys90Arg	48.3	0.994	0.211
14	chr1	150703341	HORMAD1/fs	NP_001186758.1:p.Thr327GlnfsTer18	0.1	0.954	0.716
15	chr5	23532534	PRDM9/fs	.	3.2	1.002	0.907
16	chr6	656555	HUS1B/ms	NP_683762.2:p.His130Gln	8.8	0.986	0.0732
17	chr12	101737235	SYCP3/ms	NP_001171419.1:p.Met66Thr	0.1	0.805	0.302
18	chr12	133226965	ANHX/ms	NP_001177983.1:p.Ser230Cys	18.8	1.016	0.0068
19	chr12	133227085	ANHX/ms	NP_001177983.1:p.Arg190His	0.5	1.055	0.307
20	chr14	34516452	EAPP/ms	NP_060923.2:p.Arg239Gln	3.0	1.012	0.423
21	chr19	12904533	SYCE2/ms	NP_001099048.1:p.His89Tyr	1.3	1.225	6.55×10^{-12}
22	chr20	57524058	CTCFL/ms	NP_001255969.1:p.Glu50Gln	39.4	0.999	0.911
23	chr21	43613852	HSF2BP/ns	NP_008962.1:p.Gly224Ter	0.3	1.085	0.373
24	chr22	22556814	PRAME/ms-sp	NP_001278644.1:p.Trp7Arg	41.0	0.996	0.514
25	chr22	45354086	SMC1B/ms	NP_001278430.1:p.Phe1055Leu	5.0	1.000	0.984
26	chrX	14859282	FANCB/ms	NP_001018123.1:p.Gly335Glu	7.7	0.999	0.948
**Non-coding variants**							
27	chr1	6592658	KLHL21/3'UTR	NM_001324309.2:c.*1450C>T	35.1	1.007	0.147
28	chr4	1060773	RNF212/int	NM_001366918.1:c.575-2380C>T	10.7	1.003	0.728
29	chr6	31557542	NFKBIL1/int	NM_001144961.2:c.335-86C>T	38.8	0.992	0.134
30	chr18	12945311	SEH1L/up	.	46.2	1.008	0.107
31	chr19	10582948	AP1M2/int	NM_001300887.2:c.267+658A>G	14.8	1.005	0.408
32	chr20	7328022	intergenic	.	10.4	0.985	0.0891
33	chr4	1076925	RNF212/int	NM_001131034.4:c.510+2718T>G	0.6	0.959	0.224
34	chr4	1089099	RNF212/fs	.	32.7	0.991	0.0935
35	chr14	91458683	PPP4R3A/3'UTR	NM_001284280.1:c.*76C>A	40.4	0.987	0.00939
36	chr17	44656117	MEIOC/up	.	9.7	1.004	0.542
37	chr1	150986501	ANXA9/int	NM_003568.3:c.552+86T>C	41.2	0.989	0.0194
38	chr2	54267311	ACYP2/syn	NP_001307518.1:p.Pro67=	18.6	1.002	0.755
39	chr2	96936423	FAM178B/int	NM_001122646.3:c.1079-7103C>A	40.5	1.005	0.309
40	chr11	66843352	C11orf80/3'UTR	NM_001302084.2:c.*121_*125dup	8.0	1.022	0.0131
41	chr12	133069795	intergenic	.	38.9	0.992	0.119
42	chr14	34555225	intergenic	.	0.0	1.030	0.516
43	chr20	1226073	RAD21L1/up	.	47.5	0.999	0.832
44	chr20	59864167	SYCP2/3'UTR	NM_014258.4:c.*143dup	2.9	0.963	0.0166
45	chrX	14834643	.	.	41.2	1.000	0.984
46	chrX	49297980	PPP1R3F/intergenic	.	13.5	0.992	0.255
47	chrX	151624125	PASD1/int	NM_173493.3:c.546+1061G>T	0.8	1.048	0.0405

Supplementary Note

Quality control of the SYCE2:p.His89Tyr variant rs189296436

The association analysis of rs189296436 is based on imputed rather than directly genotyped data. The imputation is based on large population specific WGS reference sets (see Methods) which allows us to reliably impute rare variants. With the exception of FinnGen the whole genome sequencing used for the imputation was performed at deCODE. The marker was not flagged for quality in any of the cohorts. Markers are flagged for quality when 1) They are in a region with > 2x average depth 2) Hardy-Weinberg equilibrium p-value < 1e-7 3) imputation information < 0.8 4) marker is reported as low quality in sequence variant calling 5) marker is in a repeat sequence 5) Sequence info < 0.6 or Sequence info > 1.4 6) Imputation yield < 70% 7) Genotyping yield < 80%. Imputation info for individual cohorts (ranging from 0.96-1.00) is reported in Supplementary Table 2. No heterogeneity was observed between cohorts for this variant.

Comparison with Laisk et al. study

In our study of pregnancy loss, we tested variants that were reported to associate with sporadic or multiple consecutive miscarriage in a study by Laisk et al. We found no association with pregnancy loss or recurrent pregnancy loss (P > 0.05) for those variants in our study. While there are many similarities between the two studies there are also differences. The main differences (described in more detail below) are:

1. The number of cases included in the current study is more than double that of the study by Laisk et al.
2. The current study defines one phenotype including sporadic and recurrent pregnancy loss while Laisk et al. attempted to define a recurrent pregnancy loss phenotype that was analyzed separately and not included in the analysis of sporadic pregnancy loss.
3. Where data was available the Laisk et al. study excluded women from the study based on age at menarche and diagnoses for conditions associated with increased susceptibility to miscarriage.

Information on pregnancy loss is mostly available either as ICD codes from electronic health records or self-reported through population specific questionnaires. While defining a single pregnancy loss is in most cases simple, defining a recurrent pregnancy loss phenotype from this type of data is more complex. This is partly because guidelines for defining recurrent pregnancy loss (European Society on

Human Reproduction and Embryology and American Society for Reproductive Medicine) have varied between two or more or three or more sporadic pregnancy losses, sometimes but not always required to be consecutive. Furthermore, data on women is often incomplete, leading to under-representation of recurrent events.

To avoid the complications inherent in the recurrent pregnancy loss phenotype we defined one pregnancy loss phenotype including women with any reported pregnancy loss, sporadic or recurrent. This resulted in a case group of 114,761 women with one or more pregnancy loss, self-reported or identified through electronic health records. The ICD codes included were ICD10:O02.1 (missed abortion), O03 (spontaneous abortion) and N96/O26.2 (recurrent pregnancy loss) as well as corresponding ICD9/8 codes.

The Laisk et al. study defined two phenotypes. Their sporadic miscarriage phenotype (N = 49,996) was limited to one or two self-reported miscarriages, or ICD-10 codes O02.1 and O03 on one or two separate time-points (at least 90 days between episodes). Their second phenotype, multiple consecutive miscarriage (N =750) was defined as follows: (i) five or more self-reported miscarriages, one live birth, no pregnancy terminations, (ii) three or more self-reported miscarriages, no live births, no pregnancy terminations, or (iii) three or more consecutive miscarriages. The first two criteria were used to ensure the consecutive nature of the miscarriages; and (iv) ICD-10 diagnosis code N96. Unlike our study they do not include ICD10 O26.2, pregnancy care for patient with recurrent pregnancy loss, in their definition of pregnancy loss cases. Other than that, the two studies use the same ICD codes to define pregnancy loss.

It is worth noting that both studies include data from the UK Biobank and based on phenotype definitions the overlap between our study and the Laisk et al. study likely extends to most of the 37,105 sporadic miscarriage cases as well as the 421 multiple consecutive miscarriage cases from the European part of the UK Biobank cohort included in their study.

Laisk et al. report one variant associating with sporadic miscarriage (rs146350366, MAF 1.2%, $P = 3.2 \times 10^{-8}$, OR = 1.4). We found no association ($P = 0.12$) between this variant and our general pregnancy loss phenotype that differs from the discovery phenotype in including recurrent pregnancy loss cases. However, given that recurrent pregnancy loss cases are only a small proportion of cases (based on both studies) it seems unlikely that this difference in phenotype definition can account for this lack of association.

Three variants were reported to associate with multiple consecutive miscarriage (rs7859844, MAF = 6.4%, $P = 1.3 \times 10^{-8}$, OR = 1.7; rs143445068, MAF = 0.8%, $P = 5.2 \times 10^{-9}$, OR = 3.4 and rs183453668, MAF = 0.5%, $P = 2.8 \times 10^{-8}$, OR = 3.8). We were unable to test rs183453668 in our data as this variant

failed in our sequencing quality control. We further note that this variant was not available in FinnGen (R8) data and it did not pass quality filters in gnomAD (gnomad.broadinstitute.org), raising concerns regarding this quality of this variant in the discovery data. We tested the remaining two variants for association in our subset of 2,253 recurrent pregnancy loss cases, and found no association ($P > 0.05$). As described above, the definition of recurrence is not the same in the two datasets. Our data is based on ICD codes only (ICD10:N96 and O26.2) while the discovery data of 750 cases only includes ICD10:N96 but in addition uses information derived from questionnaire and other data. It is, therefore, difficult to know if the same phenotype is being tested. We do note that these are low frequency variants, discovered in a small dataset with modest P-values.

Two points indicate that our pregnancy loss phenotype and sporadic pregnancy loss phenotype defined by Laisk et al. are related. We tested the genetic correlation between the two traits and found a correlation of 0.73, $P = 0.0001$. Due to sample overlap UK Biobank data was excluded from our meta-analysis in this analysis. Furthermore, the SYCE2 signal associates with sporadic miscarriage in the Laisk et al. study with $P = 5.68 \times 10^{-7}$, OR = 1.31, consistent with our results.

Reference:

Laisk, T. et al. The genetic architecture of sporadic and multiple consecutive miscarriage. Nature Communications 2020 11:1 11, 1–12 (2020).

DBDS genomics consortium:

Karina Banasik[1], Jakob Bay[2], Jens Kjærgaard Boldsen[3], Thorsten Brodersen[2], Søren Brunak[1], Kristoffer Burgdorf[1], Mona Ameri Chalmer[4], Maria Didriksen[5], Khoa Manh Dinh[3], Joseph Dowsett[5], Christian Erikstrup[3,6], Bjarke Feenstra[5,7], Frank Geller[5,7], Daniel Gudbjartsson[8], Thomas Folkmann Hansen[4], Lotte Hindhede[3], Henrik Hjalgrim[9,7], Rikke Louise Jacobsen[5], Gregor Jemec[10], Bitten Aagaard Jensen[11], Katrine Kaspersen[3], Bertram Dalskov Kjerulff[3], Lisette Kogelman[4], Margit Anita Hørup Larsen[5], Ioannis Louloudis[1], Agnete Lundgaard[1], Susan Mikkelsen[3], Christina Mikkelsen[5], Ioanna Nissen[5], Mette Nyegaard[12], Sisse Rye Ostrowski[5,13], Ole Birger Pedersen[2,13], Alexander Pil Henriksen[1], Palle Duun Rohde[12], Klaus Rostgaard[9,7], Michael Schwinn[5], Kari Stefansson[8], Hreinn Stefansson[8], Erik Sørensen[5], Unnur Thorsteinsdottir[8], Lise Wegner Thørner[5], Mie Topholm Bruun[14], Henrik Ullum[15], Thomas Werge[13], David Westergaard[1]

[1]Novo Nordisk Foundation Center for Protein Research, Faculty of Health and Medical Sciences, University of Copenhagen, Copenhagen, Denmark
[2]Department of Clinical Immunology, Zealand University Hospital, Køge, Denmark
[3]Department of Clinical Immunology, Aarhus University Hospital, Aarhus, Denmark
[4]Danish Headache Center, Department of Neurology, Copenhagen University Hospital, Rigshospitalet-Glostrup, Copenhagen, Denmark

[5]Department of Clinical Immunology, Copenhagen University Hospital, Rigshospitalet, Copenhagen, Denmark
[6]Department of Clinical Medicine, Health, Aarhus University, Aarhus, Denmark
[7]Department of Epidemiology Research, Statens Serum Institut, Copenhagen, Denmark
[8]deCODE genetics/Amgen, Inc., Reykjavik, Iceland
[9]Danish Cancer Society Research Center, Copenhagen, Denmark
[10]Department of Dermatology, Zealand University hospital, Roskilde, Denmark
[11]Department of Clinical Immunology, Aalborg University Hospital, Aalborg, Denmark
[12]Department of Health Science and Technology, Faculty of Medicine, Aalborg University, Aalborg, Denmark
[13]Department of Clinical Medicine, Faculty of Health and Medical Sciences, University of Copenhagen, Copenhagen, Denmark
[14]Department of Clinical Immunology, Odense University Hospital, Odense, Denmark
[15]Statens Serum Institut, Copenhagen, Denmark
