# Supplementary material for: Variant in the synaptonemal complex protein SYCE2 associates with pregnancy loss through effect on recombination
Source: Nat Struct Mol Biol. 2024 Jan 29;31(4):710–6. doi: 10.1038/s41594-023-01209-y (PMC11026158; doi:10.1038/s41594-023-01209-y)
Supplement: Supplementary file 2 — Reporting Summary [file 41594_2023_1209_MOESM2_ESM.pdf]

nature portfolio

Corresponding author(s): V. Steinthorsdottir and K. Stefansson

Last updated by author(s): Nov 29, 2023

Reporting Summary

Nature Portfolio wishes to improve the reproducibility of the work that we publish. This form provides structure for consistency and transparency in reporting. For further information on Nature Portfolio policies, see our Editorial Policies and the Editorial Policy Checklist.

Statistics

For all statistical analyses, confirm that the following items are present in the figure legend, table legend, main text, or Methods section.

n/a	Confirmed	
☐	☒	The exact sample size (n) for each experimental group/condition, given as a discrete number and unit of measurement
☒	☐	A statement on whether measurements were taken from distinct samples or whether the same sample was measured repeatedly
☐	☒	The statistical test(s) used AND whether they are one- or two-sided<br>*Only common tests should be described solely by name; describe more complex techniques in the Methods section.*
☐	☒	A description of all covariates tested
☐	☒	A description of any assumptions or corrections, such as tests of normality and adjustment for multiple comparisons
☐	☒	A full description of the statistical parameters including central tendency (e.g. means) or other basic estimates (e.g. regression coefficient) AND variation (e.g. standard deviation) or associated estimates of uncertainty (e.g. confidence intervals)
☐	☒	For null hypothesis testing, the test statistic (e.g. F, t, r) with confidence intervals, effect sizes, degrees of freedom and P value noted<br>*Give P values as exact values whenever suitable.*
☒	☐	For Bayesian analysis, information on the choice of priors and Markov chain Monte Carlo settings
☒	☐	For hierarchical and complex designs, identification of the appropriate level for tests and full reporting of outcomes
☒	☐	Estimates of effect sizes (e.g. Cohen's d, Pearson's r), indicating how they were calculated

Our web collection on statistics for biologists contains articles on many of the points above.

Software and code

Policy information about availability of computer code

Data collection	No software was used for data collection
Data analysis	R (version 4.2.2, lm version 4.2.2), (version 3.3.3, xoi – version 0.67-1) (3.6.3, glmmTMB version 1.1.7), python 3.8.1 (and packages numpy 1.24.2, pandas 1.4.0, scipy 1.10.1, statsmodels 0.13.2)

For manuscripts utilizing custom algorithms or software that are central to the research but not yet described in published literature, software must be made available to editors and reviewers. We strongly encourage code deposition in a community repository (e.g. GitHub). See the Nature Portfolio guidelines for submitting code & software for further information.

Data

Policy information about availability of data

All manuscripts must include a data availability statement. This statement should provide the following information, where applicable:

- Accession codes, unique identifiers, or web links for publicly available datasets
- A description of any restrictions on data availability
- For clinical datasets or third party data, please ensure that the statement adheres to our policy

GRCh38.p1: https://www.ncbi.nlm.nih.gov/datasets/genome/GCF_000001405.27/. FinnGen summary statistics were obtained at https://www.finngen.fi/en/access_results. The GWAS summary statistics for the pregnancy loss meta-analysis are deposited at https://www.decode.com/summarydata/

Research involving human participants, their data, or biological material

Policy information about studies with human participants or human data. See also policy information about sex, gender (identity/presentation), and sexual orientation and race, ethnicity and racism.

Reporting on sex and gender	The GWAS meta-analysis included females with reported pregnancy loss and female population controls. Analysis of recombination traits included males and females.
Reporting on race, ethnicity, or other socially relevant groupings	Individuals included in the study were of European origin.
Population characteristics	The mean birth year of Icelandic cases was 1949 (interquartile range (IQR) 1930-1965) and controls 1970 (IQR 1950-2000). Mean birth year of Danish cases was 1970 (IQR 1960-1980) and controls 1974 (IQR 1964-1986). Mean birth year of UK cases was 1952 (IQR 1945-1958) and controls 1951 (IQR 1945-1957). Mean birth year of USA cases was 1980 (IQR 1974-1987) and controls 1961 (IQR 1947-1977).
Recruitment	For each contributing study, individuals with the following ICD codes, (ICD10:O03; ICD9;634; ICD8:643), (ICD10:N96, O262; ICD9:6298; ICD8:6430), or (ICD10:O021; ICD9:632; ICD8:634, 6451) or self reported pregnancy loss were included as cases. For each study, the control group consisted of females not included in the case group.
Ethics oversight	The Icelandic study was approved by the Icelandic National Bioethics Committee. The CHB Study was approved by the National Committee on Health Research Ethics and the Capital Region Data Protection Agency. The DBDS study was approved by the Danish National Committee on Health Research Ethics and the Capital Region Data Protection Agency. The North West Research Ethics Committee approved UK Biobank study. The Intermountain Healthcare Institutional Review Board approved the USA study. The Coordinating Ethics Committee of the Helsinki and Uusimaa Hospital District approved the FinnGen research project.

Note that full information on the approval of the study protocol must also be provided in the manuscript.

Field-specific reporting

Please select the one below that is the best fit for your research. If you are not sure, read the appropriate sections before making your selection.

☒ Life sciences ☐ Behavioural & social sciences ☐ Ecological, evolutionary & environmental sciences

For a reference copy of the document with all sections, see nature.com/documents/nr-reporting-summary-flat.pdf

Life sciences study design

All studies must disclose on these points even when the disclosure is negative.

Sample size	For the GWAS meta-analysis we combined the largest samples size of pregnancy loss phenotypes available within the contributing studies.
Data exclusions	For each contributing study samples and variants were excluded based on well established sample and variant quality control procedures to remove poor quality samples and variants.<br>For the GWAS meta-analysis variants with minor allele frequency < 0.01% or imputation info < 0.8 in all cohorts, as well as variants with discrepant allele frequency between cohorts, were further excluded.
Replication	The GWAS meta-analysis included all study material for the relevant phenotypes available to us so we did not conduct replication. There was no evidence for heterogeneity between studies indicating that the results were not driven by a single false positive study.
Randomization	The case group comprised all women who fulfilled the diagnostic criteria and the control group comprise all women who did not fulfill the diagnostic criteria. No randomization was applied.
Blinding	This is an observational study and no blinding was required

Reporting for specific materials, systems and methods

We require information from authors about some types of materials, experimental systems and methods used in many studies. Here, indicate whether each material, system or method listed is relevant to your study. If you are not sure if a list item applies to your research, read the appropriate section before selecting a response.

Materials & experimental systems

n/a	Involved in the study
☒	☐ Antibodies
☒	☐ Eukaryotic cell lines
☒	☐ Palaeontology and archaeology
☒	☐ Animals and other organisms
☒	☐ Clinical data
☒	☐ Dual use research of concern
☒	☐ Plants

Methods

n/a	Involved in the study
☒	☐ ChIP-seq
☒	☐ Flow cytometry
☒	☐ MRI-based neuroimaging
